# Enhancing biomass conservation and enzymatic hydrolysis of sweet sorghum bagasse by combining pretreatment with ensiling and NaOH

**DOI:** 10.3389/fmicb.2024.1370686

**Published:** 2024-03-15

**Authors:** Shuai Zhao, Hanyan Li, Tawatchai Sumpradit, Aman Khan

**Affiliations:** ^1^School of Bioengineering, Henan University of Technology, Zhengzhou, China; ^2^Microbiology and Parasitology Department, Naresuan University, Phitsanulok, Thailand; ^3^Pakistan Agricultural Research Council, Islamabad, Pakistan

**Keywords:** sweet sorghum bagasse, *Lactobacillus plantarum*, ensiling enzyme, NaOH pretreatment, enzymatic hydrolysis

## Abstract

Lignocellulosic pretreatment is an important stage in biomass utilization, which usually requires high input. In this study, a low-cost method using combined ensiling and NaOH was developed for lignocellulosic pretreatment. Sweet sorghum bagasse (SSB) was ensiled for 21 days and then treated with diluted NaOH (0%, 1%, and 2%) for fermentation. The results showed that the application of *Lactobacillus plantarum* (L) reduced fermentation losses of the silages, mainly low water-soluble carbohydrate (WSC) and ammonia nitrogen loss. Meanwhile, the application of *Lactobacillus plantarum* and ensiling enzyme (LE) promoted lignocellulosic degradation, as evidenced by low neutral detergent fiber (NDF), acid detergent fiber (ADF), lignin (ADL), and hemicellulosic (HC) contents. The dominant bacterial genera were *Lactobacillus, uncultured_bacterium_f_Enterobacteriaceae*, and *Pantoea* after silage, which corresponded to the higher lactic acid and acetic contents and lower pH. The reducing sugar yields of SSB increased after combined pretreatment of silage and NaOH and were further enhanced by the 2% NaOH application, as evidenced by the high reducing sugar yield and microstructure damage, especially in the L-2% NaOH group and the LE-2% NaOH group, in which the reducing sugar yields were 87.99 and 94.45%, respectively, compared with those of the no additive control (CK)-0 NaOH group. Therefore, this study provides an effective method for SSB pretreatment to enhance biomass conservation.

## 1 Introduction

The demand for fossil fuels is rising annually due to the rapid social and economic development, which leads to a number of environmental issues like global warming. In recent years, biomass has been recognized as favored energy due to its advantages of sustainability and carbon emission reduction (Kumar et al., 2022; Caldera et al., 2023). Sweet sorghum bagasse (SSB) is a short perennial crop that can be adapted to almost all climates. Sweet sorghum, with its starchy grain and sugar-rich stem growing at a rapid rate and possessing unique characteristics, contains cellulose, hemicellulose, and lignin in proportions of 27%−44.6%, 25%−27.1%, and 11%−24.7%, respectively (Appiah-Nkansah et al., 2019). These qualities make sweet sorghum a viable candidate as an energy crop, as it can be readily fermented by *Saccharomyces cerevisiae* to produce alcohol (Erbetta et al., 2024). The lignocellulosic fraction that remains after the extraction of juice, specifically sweet sorghum bagasse (SSB), serves as a non-edible feedstock for biofuel production (Khalili and Amiri, 2020). However, the low biodegradability and bioavailability of SSB limited its application. Additionally, the seasonality and timeliness of the harvest of sweet sorghum cannot be ignored. Thus, it is necessary to develop appropriate storage methods to ensure the sustainable supply and effective storage of SSB.

Pretreatment can decompose or remove lignin and degrade hemicellulose by changing the microstructure, macrostructure, and chemical composition of materials, changing cellulose crystallinity, increasing the contact area of enzymes, and improving the efficiency of cellulose hydrolysis (Niju and Swathika, 2019; Tinôco et al., 2021; Wang et al., 2021). Traditional pretreatment methods for lignocellulosic biomass included physical (mechanical), chemical (acid and alkali), and biological (mainly enzymes, fungal, and microbial) pretreatments (Cai et al., 2021; Sun et al., 2021). Among them, silage is an effective biological method that can supply raw materials for long-term bioenergy production (Li F. et al., 2019; Li P. et al., 2019; Li F. H. et al., 2021; Li H. et al., 2021; Li J. et al., 2021). The ensiling process can not only retain more than 90% of the plant energy but also improve the effect of enzymatic hydrolysis (Dewar, 1963). A previous study reported that incorporating *Lactobacillus plantarum* into sorghum silage can enhance fermentation quality, lignocellulose degradation, and enzymatic saccharification (Usman et al., 2022). Nevertheless, silage pretreatment is not without its limitations, including extended pretreatment cycles and suboptimal efficiency in lignin degradation (Wang T. et al., 2020).

Recently, alkali pretreatment achieved by NaOH is the most widely used and effective method for various lignocelluloses to enhance their cellulosic utilization through addressing textural recalcitrance (Wang et al., 2021). NaOH pretreatment can destroy the ester and ether bonds of lignin and increase the porosity of the biomass (Rezania et al., 2020; Zhang H. et al., 2020; Zhang J. et al., 2020; Wang et al., 2021). In addition, NaOH can also dissociate into Na^+^ and OH^−^ to further promote the hydrolysis rate (Tang et al., 2023). Zhang reported that the lignin content of bamboo's outer and inner layers was extremely decreased from 22.2 and 21.4% to 10.1% and 10.4 with 8% (w/v) NaOH pretreatment. In another investigation, a 7.5% (w/v) NaOH solution was utilized for the pretreatment of corn stover, leading to a notable 37.1% increase in lignin removal, as observed in the Results section (You et al., 2019). All these results revealed that the alkali pretreatment could effectively promote the dissolution of lignin and hemicellulose, improving lignin dispersion and breaking the ester bond (Wang W. et al., 2020). However, the use of large quantities of alkali reagents increases the cost of lignocellulosic biorefinery. Therefore, it is difficult for NaOH pretreatment alone to fit into the strategic thought of sustainable development.

In this study, we propose using the combined pretreatment of ensiling and NaOH to improve the sustainable supply and quality storage of SSB. The effects of different additives (ensiling enzyme, *L. plantarum*, and ensiling enzyme + *L. plantarum*) on the fermentation quality and nutritional components of SSB silage were observed. The microstructure and enzymatic hydrolysis of SSB were also investigated. To the best of our knowledge, this is the first time the application of ensiling enzyme combined with NaOH in silage fermentation has been reported, which could provide a high-efficiency and low-cost pretreatment technology.

## 2 Methods and materials

### 2.1 Bio-pretreatment by ensiling process

The whole-plant sweet sorghum was taken from the sorghum experimental field (N 36.55°latitude, E 104.18° longitude, altitude 1,724.76 m a.s.L., Baiyin, China) of the Institute of Modern Physics, Chinese Academy of Sciences, Lanzhou, China. The stalks were squeezed by a three-roller mill to obtain the liquid and bagasse separately (Cao et al., 2012). The bagasse was cut to a size of 1–2 cm, dried at 65°C, and then ground to pass through a sieve with 100 meshes. Finally, it was stored in a plastic bag at room temperature. *L. plantarum* (ACCC 11016) was purchased from the China Agricultural Microorganism Culture Collection and Management Center. It was inoculated and cultured in de Man, Rogosa, and Sharpe (MRS) broth medium, according to the previous study (Zhao et al., 2018). After incubation, the concentration of *L. plantarum* was determined by the plate counting method. The ensiling enzyme (composed of cellulase, hemicellulase, glucanase, pectinase, and protease) was purchased from Ningxia Heshibi Biotechnology Co., Ltd. (Ningxia, China), and the enzyme activity was >50,000 U/g.

The treatment procedures were as follows: (i) no additive control (CK), 5 ml/g of distilled water on a fresh matter basis; (ii) *L. plantarum* (L) at a recommended application rate of 10^8^ cfu/g FM, as described by Zhao et al. (2018); (iii) ensiling enzyme (E), at an optimal rate of 5% FM; and (iv) *L. plantarum* + ensiling enzyme (LE). Thereafter, chopped SSB was mixed homogeneously with each additive, packed manually into 30 cm × 45 cm vacuum packaging bags, and stored at the ambient temperature (25 ± 3°C) after being packed. A total of three bags for each treatment were opened after 21 days of ensiling. A total of 12 samples (four treatments × three replicates) were prepared for the analysis of fermentation quality, chemical composition, bacterial community composition, and NaOH pretreatment.

### 2.2 NaOH pretreatment after ensiling pretreatment

All the samples were treated at 50°C for 4 h by 1 and 2% (w/v) NaOH solutions with a solid-liquid ratio of 1:10 (w/v), respectively. The pretreatment was performed in a 250-ml conical flask in a constant-temperature shaker with a rotation speed of 200 rpm. After the pretreatment, the slurry was filtered to separate out the solid residue and washed with water several times until its pH values were around 7.0. The solid residue was dried to a constant mass in an oven at 105°C and placed in a desiccator at room temperature for micro-structural observation and enzymatic scarification.

### 2.3 Chemical composition analysis

Another part of the sample was dried at 65°C, crushed at a constant weight, and then passed through a 100-mesh sieve for analysis of composition. The dry matter (DM) percentage was determined by the 105°C drying constant weight method (<0.01 g). The content of total nitrogen (TN), WSC (water-soluble carbohydrates), starch (ST), acid detergent fiber (ADF), neutral detergent fiber (NDF), and acid detergent lignin (ADL) was quantified using the methods of previous studies (Chen et al., 2020; Meng et al., 2023). Hemicellulose (HC), cellulose (CL), and hemicellulose (HoC) contents were calculated as described in the previous study (Ren et al., 2021). The crude protein (CP) content was calculated as TN × 6.25. The silage pretreatment effect was evaluated by the membership function value. There were 10 silage pretreatment traits, including negative correlation indexes (ADF, NDF, ADL, pH, and AN) and positive correlation indexes (LA, AA, WSC, CP, and DM). The modified membership function value of the silage pretreatment effect was calculated following the equations (Chen et al., 2012; Wang et al., 2022):


(1) $$\begin{array}{l} \text{y}\left(\text{xi}\right)\frac{\text{xi}-x\min}{x\max-x\min} \end{array}$$

(2) $$\begin{array}{l} \text{y}\left(xi\right)=1-\frac{xi-x\min}{x\max-x\min} \end{array}$$

where *y*(*xi*) is the membership function value of the trait; *x*max is the maximum value of the index coefficient for the trait; and *x*min is the minimum value of the index coefficient for the trait. If the measured indexes were positively correlated with the silage pretreatment effect, then Equation (1) was used for calculation. Equation (2) was selected for the negative correlation calculation.

### 2.4 Fermentation quality analysis

To analyze the fermentation quality, a 50-g fresh sample was homogenized in 450 ml of distilled water by a juice extractor for 2 min. The homogenized mixture was filtered through four layers of medical gauze, and the filtrate was used for the analysis of ensiling fermentation characteristics, including pH, organic acids (lactic, acetic, propionic, and butyric acids), and ammonia nitrogen (AN) as described by Meng et al. (2023).

### 2.5 Micro-structural observation of silages by SEM and FTIR

Scanning electron microscopy (SEM) (TESCAN MIRA3, Shanghai, China) was used to observe the microstructure of sweet sorghum bagasse. The sample was evenly smeared on the double-sided conductive adhesive, and after marking, a layer of conductive metal was sprayed on the surface of the sample with a gold sprayer at a current of 10 mA, and the gold spraying time was 90 s. The gold-sprayed samples were placed on the SEM stage and scanned at an accelerating voltage of 8 kV to obtain pictures. The dry sweet sorghum bagasse sample was mixed with KBr in a ratio of 1:100 and pressed into slices, and the pure KBr was ground and pressed as the scanning background for scanning and analysis by a FTIR spectrometer (YP-3, Shanghai, China). The scanning wavelength range is 500–4,000 cm^−1^, the scanning resolution is 4 cm^−1^, and the scanning is performed 60 times per second (Xu et al., 2019). The total crystallinity index (TCI) was defined as the ratio of the absorbance at 1,375 to 2,900 cm^−1^ (Ostovareh et al., 2015).

### 2.6 Microbial community analysis

Bacterial community analysis of silage was carried out at Beijing Baimaike Biotechnology Co., Ltd. (Beijing, China) by high-throughput sequencing. DNA was extracted from the samples using a TGuide S96 soil/feces DNA kit (Tiangen Biochemical Technology Limited, Beijing, China) in accordance with the kit's instructions. The DNA obtained from each sample was subjected to two-step PCR amplification to construct a small-fragment sequencing library. For the first amplification step, the 16S rRNA gene of V3–V4 was amplified by extracting DNA as a template (primers: 338F, 5′-ACTCCTACGGGAGGCAGCA-3′; 806R, 5′-GGACTACHVGGGTWTCTAAT-3′). The PCR conditions were as follows: 95°C for 5 min, 95°C for 30 s, 50°C for 30 s, 72°C for 40 s, and 72°C for 7 min for a total of 25 cycles. The second round of PCR amplification was performed under the following conditions: 98°C for 30 s; 98°C for 10 s, 65°C for 30 s, and 72°C for 30 s for a total of 10 cycles; 72°C for 5 min. The PCR products were recycled from a 1.8% agarose gel and purified using an OMEGA DNA purification column. The purified products were quantified using a Quant-iT PicoGreen dsDNA assay kit in accordance with the kit's instructions. Thereafter, the amplicons were sequenced on an Illumina Novaseq 6000 sequencing platform using the QIIME2 software. The alpha diversity index was calculated using the Mothur software (version 1.9.1), and the abundance at the phylum level and the genus level were displayed with the Origin software (version 2021) to evaluate the bacterial community compositions. A heat map was drawn based on the correlation between bacterial genus level, fermentation parameters, and chemical components.

### 2.7 Statistical analysis

The data displayed are the means ± standard deviation. A one-way analysis of variance (ANOVA) was used to analyze the differences between the treatments, and Dunnett's multiple comparisons were used to analyze the significance. Data were considered significant when the *p*-value was <0.05. The data were analyzed using the SPSS 20 and Origin 2021 (Originlab, United States) software.

## 3 Results and discussion

### 3.1 Enzymatic hydrolysis after silage and NaOH combined pretreatment

**Figure 1:**
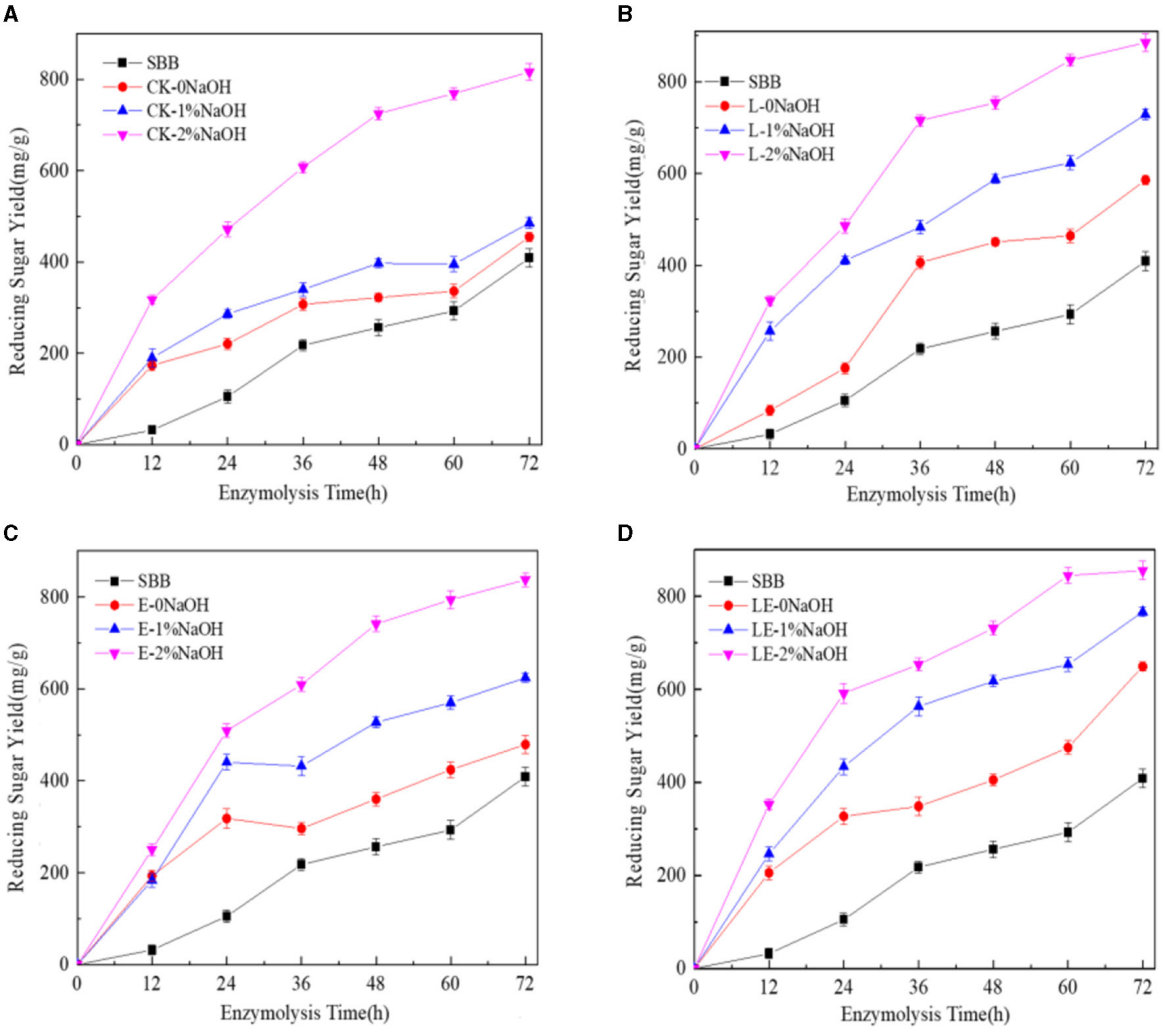
Yield of reducing sugar after enzymatic hydrolysis by combined pretreatment. CK, no additive control; L, *Lactobacillus plantarum*; E, ensiling enzyme; LE, *L. plantarum*+ensiling enzyme. **(A)** Combined pretreatment of ensiling without addition and different concentrations of NaOH. **(B)** Combined pretreatment of *L. plantarum* ensiled with different concentrations of NaOH. **(C)** Combined pretreatment of ensiling enzyme ensiled with different concentrations of NaOH. **(D)** Combined pretreatment of *L. plantarum*+ensiling enzyme ensiled with different concentrations of NaOH.

**Figure 2:**
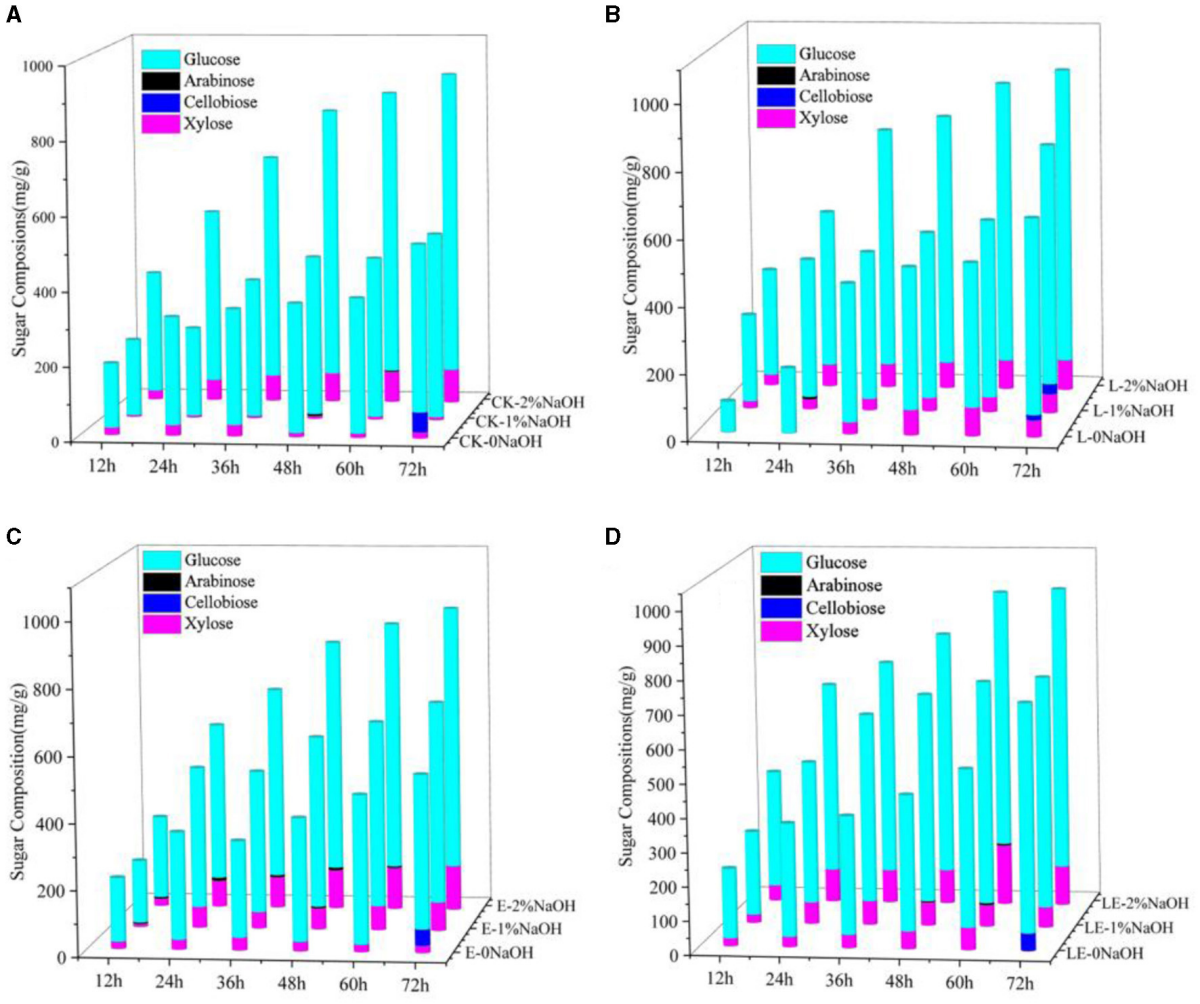
Monosaccharide components after enzymatic hydrolysis by combined pretreatment. CK, no additive control; L, *Lactobacillus plantarum*; E, ensiling enzyme; LE, *L. plantarum*+ensiling enzyme. **(A)** Combined pretreatment of ensiled without addition and different concentrations of NaOH. **(B)** Combined pretreatment of *L. plantarum* ensiled with different concentrations of NaOH. **(C)** Combined pretreatment of ensiling enzyme ensiled with different concentrations of NaOH. **(D)** Combined pretreatment of *L. plantarum*+ensiling enzyme ensiled with different concentrations of NaOH.

The enzymatic hydrolysis of SBB after the combined pretreatment of silage and NaOH was tested. As shown in Figure 1, the yield of reducing sugar in the combined pretreatment group of silage and NaOH was much higher than that of the single silage treatment group in any enzymatic hydrolysis time period, indicating that the combined pretreatment effect of silage and NaOH was better than that of single silage pretreatment. After 72 h of enzymatic hydrolysis, the yield of reducing sugar in the E-2%NaOH group was 837.47 mg/g, and the yields of reducing sugar in the L-2%NaOH group and LE-2%NaOH group were 885.15 and 855.73 mg/g, respectively. After the combined pretreatment of silage and 2% NaOH, the yield of reducing sugar in each treatment group was the highest. In the low concentration range, the better pretreatment effect was obtained at the higher NaOH concentration. Among all treatment groups, the yields of reducing sugar in the L-2%NaOH group and the LE-2%NaOH group were as high as 885.15 and 855.73 mg/g. Furthermore, glucose and xylose were found to be mainly enzymatic hydrolysis, and the change trend of xylose yield was similar to the change trend of glucose yield, but the xylose yield in all treatment groups was much lower than the glucose concentration (Figure 2). In conclusion, both silage and NaOH treatment can improve the yield of reducing sugar, and the enzymatic hydrolysis of silage and 2% NaOH combined pretreatment had the highest yield of reducing sugar and the best enzymatic hydrolysis effect.

A previous study reported that the optimal concentration of NaOH for enzymatic hydrolysis was between 3 and 5% (Yan et al., 2020). It was also found that, when 2% NaOH was used to pretreat SSB, the enzymatic hydrolysis was more effective than 10% H_2_O_2_ and 2% H_2_SO_4_ (Dong et al., 2019). In this study, the yields of reducing sugar in the L-2%NaOH group and the LE-2%NaOH group were as high as 885.15 and 855.73 mg/g, which was much higher than a previous report in which the highest yield of reducing sugar was 750 mg/g after enzymatic hydrolysis for sweet sorghum bagasse (Mishra et al., 2017). After 72 h of enzymatic hydrolysis, there was a small amount of cellobiose in the silage group, while there was a small amount of arabinose in the silage and NaOH combined treatment group. After enzymatic hydrolysis, the monosaccharides in the enzymatic hydrolysate of all treatment groups were mainly glucose and xylose, and the change trend of xylose yield was similar to the change trend of glucose yield, but the xylose yield in all treatment groups was much lower than the glucose concentration, which is because silage and NaOH pretreatment caused partial degradation of hemicellulose and lignin, which increased the porosity of cellulose and allowed hydrolase to penetrate, thereby increasing glucose production, which was consistent with the SEM results and also consistent with the previous report (Cao et al., 2012). This difference was related to the addition of different enzymes in the enzymatic hydrolysis process, as well as different raw materials.

### 3.2 SEM analysis after silage and NaOH-combined pretreatment

**Figure 3:**
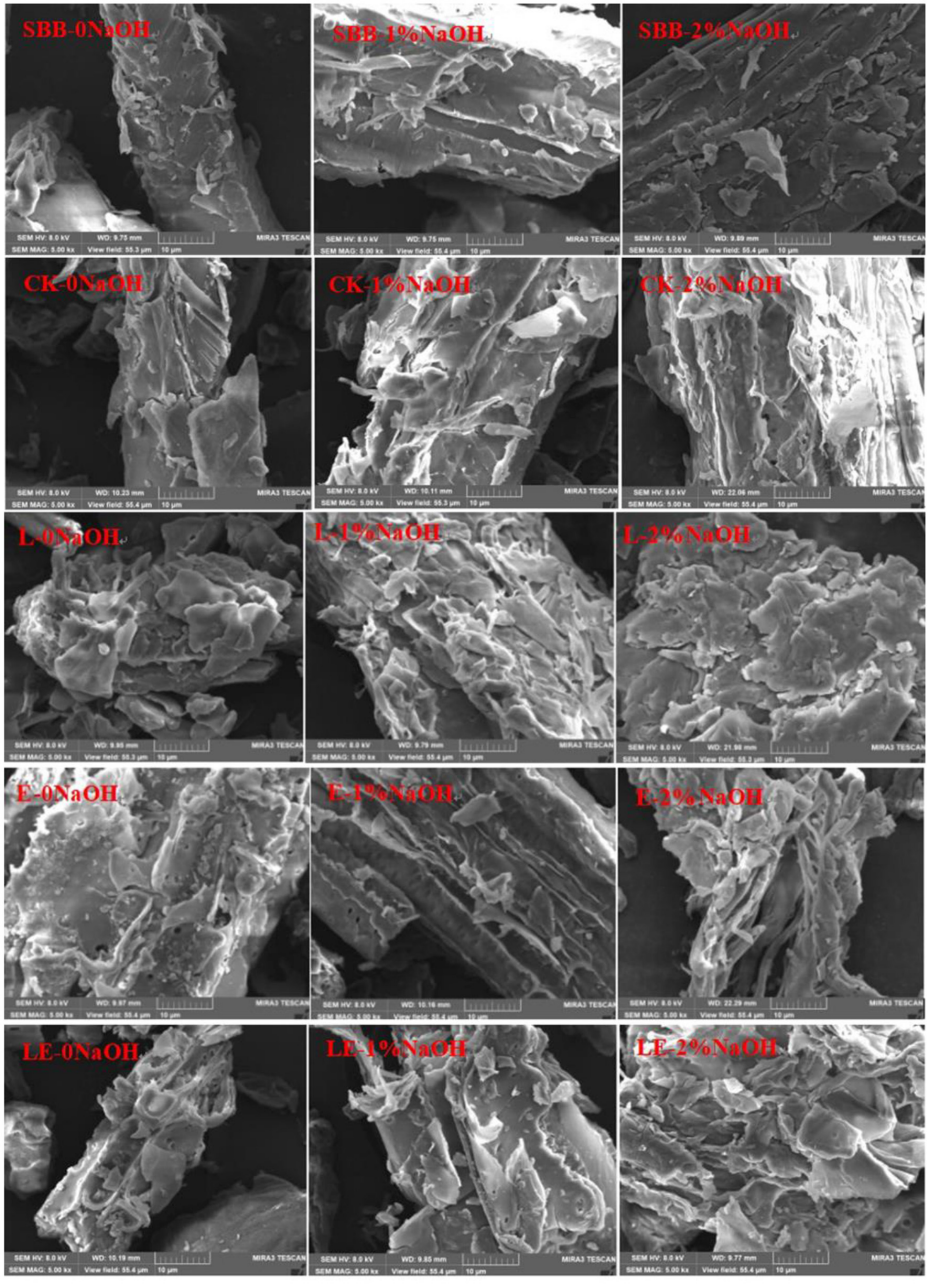
SEM of sweet sorghum bagasse pretreated with silage and NaOH. SBB, sweet sorghum bagasse; CK, no additive control; L, *Lactobacillus plantarum*; E, ensiling enzyme; LE, *L. plantarum*+ensiling enzyme.

The accessible surface area of lignocellulose consists of an external specific surface area and an internal specific surface area. External specific surface area can be roughly assessed by scanning electron microscope (SEM) images. As shown in Figure 3, the raw material of SSB has a dense structure. After silage pretreatment, the surface of the SSB in the four treatment groups was damaged to a certain extent. After the mass concentration NaOH solution treatment, the surface of all the samples in the combined biochemical treatment group formed a loose structure, and a large number of fractures and stratifications appeared. Moreover, with the increase in NaOH solution concentration, the degree of structural damage to sweet sorghum bagasse became more serious. The surface of all silage pretreatment groups was damaged, which may be caused by the destruction of sweet sorghum structure during the pressing process and also related to the degradation of carbohydrates during the silage process. The damage degree of the surface structure of the L-0NaOH group and the LE-0NaOH group was more serious than that of the CK-0NaOH group and the E-0NaOH group, indicating that adding *L. plantarum* and cyanobacteria silage enzymes during silage pretreatment was conducive to the degradation of lignocellulose. In addition, the damage degree to the surface structure of all silage and NaOH combined treatment groups was more serious than that of the single silage group. It could be observed that the surface structure of SSB was seriously damaged, and the surface was full of scattered fiber fragments with holes and cracks; most of the cell shape of cellulose was dissolved; and more surface collapse was visible. It was also found that SSB treated with 2%NaOH for 2 h could destroy the cell wall of sweet sorghum bagasse, increase the surface area, and make it easier for enzymatic hydrolysis.

A previous study reported that SEM can be used to observe the morphology of materials. In the present study, the morphology of SBB was significantly changed after combined pretreatment when compared to raw SBB. Similar results were also observed in a previous study reported by Li et al. (2020). This morphological change is caused by the saponification reaction between NaOH and the intermolecular ester bond of lignin, which results in the removal of the surface lignin layer and the release of cellulose from the biomass matrix, exposing more of the internal structure and increasing the external area (Umagiliyage et al., 2015). Additionally, Dong et al. also found that SEM images of NaOH-pretreated SSB showed a looser porous structure and special surface area, which greatly increased the accessibility of cellulose to biomass (Dong et al., 2019). In conclusion, it can be seen intuitively from the scanning electron microscope photos that the combined pretreatment of silage and NaOH can expose cellulose in SSB and make cellulose more easily exposed to acids and enzymes, thus improving the hydrolysis efficiency of lignocellulose.

### 3.3 FTIR analysis after silage and NaOH combined pretreatment

**Figure 4:**
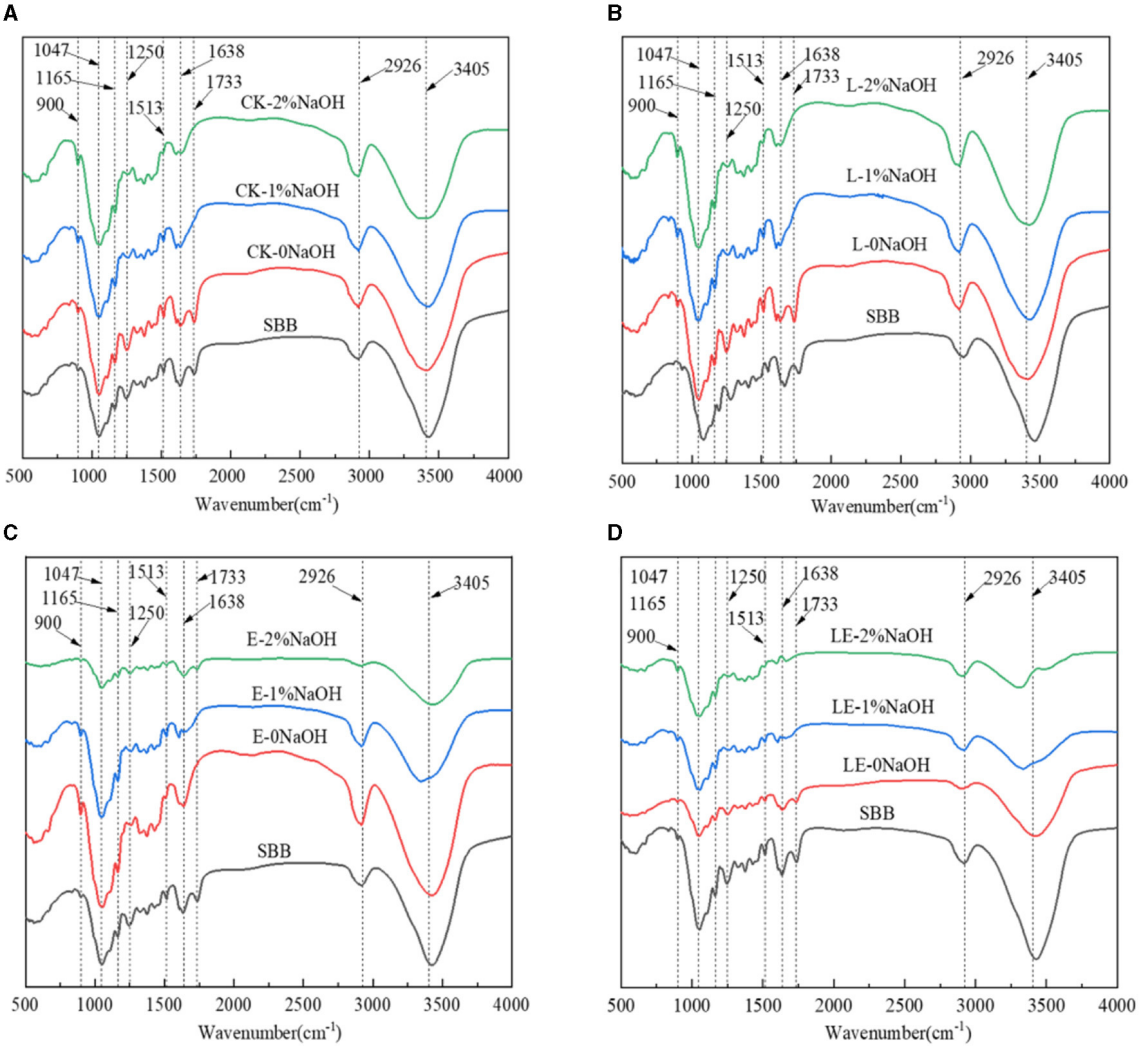
FT-IR spectra of sweet sorghum residue pretreated with silage and NaOH. SBB, sweet sorghum bagasse; CK, no additive control; L, *Lactobacillus plantarum*; E, ensiling enzyme; LE, *L. plantarum*+ensiling enzyme. **(A)** Combined pretreatment of ensiling without addition and different concentrations of NaOH. **(B)** Combined pretreatment of *L. plantarum* ensiled with different concentrations of NaOH. **(C)** Combined pretreatment of ensiling enzyme ensiled with different concentrations of NaOH. **(D)** Combined pretreatment of *L. plantarum*+ensiling enzyme ensiled with different concentrations of NaOH.

To further investigate the specific effects of silage combined with pretreatment with NaOH on the chemical structure of lignocellulose from SSB, FTIR analysis of the treated samples in the range of 500–4,000 cm^−1^ was conducted. As can be observed from Figure 4, the locations of absorption peaks in all groups were basically the same, occurring at ~3,405, 2,926, 1,733, 1,638, 1,515, 1,250, 1,165, 1,047, and 900 cm^−1^. Compared with the raw material of SSB, the peak at 1,733 cm^−1^ disappeared after combined pretreatment, which may be the result of lignin and partial hemicellulose being removed by NaOH. On the other hand, after the combined pretreatment of silage and NaOH, the characteristic peaks at 1,638 cm^−1^ in all the combined treatment groups were weakened, and the peak at 1,638 cm^−1^ was mainly caused by C=C stretching of the aromatic ring of lignin. It was also reported that, after the combined pretreatment of syringic acid and gallic acid on SSB, the peak intensity decreased significantly to 1,638 cm^−1^. After combined pretreatment, the characteristic peaks at 1,513 and 1,250 cm^−1^ in all groups were weakened, indicating that the delignification effect was very obvious after the combined pretreatment of silage and NaOH, and the content of lignin was reduced significantly. At 1,165 and 1,047 cm^−1^, the peak shapes of all combined pretreatment groups changed, which may be caused by the change in lignocellulose structure. In addition, the peak at 900 cm^−1^ represents the β-glycosidic bond.

FTIR is a commonly used technology for functional group analysis of materials before and after treatment. Several previous studies have reported using FTIR for functional material analysis, like biochar and metal materials. The FTIR analysis of the SBB before and after treatment in this investigation revealed a variety of findings. The FTIR of ensiling groups mainly showed the changes of peak shape strength at some wavelengths, while NaOH treatment not only showed changes in peak shape strength but also the summit disappeared. The results showed that for the single silage group, the peak of the E-0NaOH group and the LE-0NaOH group at 1,733 cm^−1^ was significantly weaker than that of raw material and other groups, and the peak here was the characteristic peak of the lignin-hemicellulose complex, indicating that ensiling enzyme alone or synergistically with *L. plantarum* can degrade part of hemicellulose. In addition, the peak of the four silage treatment groups at 1,250 cm^−1^ was weaker than that of the SBB group, indicating that silage pretreatment had a great influence on the C=O bond of lignin. The peak intensity of all combined pretreatment groups was greater than that of the SBB group and the silage pretreatment group alone, indicating that the combined pretreatment of silage and NaOH leads to partial cleavage of glycosidic bonds in cellulose and hemicelluloses, which is consistent with the previous research results (Wu et al., 2020). The pretreatment results of crystallinity index showed similar results to a previous study, which resulted in a reduction of CI and TCI from 0.871 and 1.72 for untreated stalks to 0.656 and 1.53 for stalks pretreated with 50% ethanol at 160°C and 0.799 and 1.46 for stalks pretreated with 50% ethanol at 140°C, respectively (Ostovareh et al., 2015). Moreover, the technoeconomic analysis in comparison with several previous studies is performed in Table 1. These results demonstrated that combined treatment using silage and NaOH was more effective and low-cost than treatment alone.

**Table 1:** The technoeconomic analysis of the present study.

**Pretreatment method**	**Cost (normalized to one reaction)**	**References**
Dilute NaOH solution autoclaving pretreatment	0.225/kg	Cheng et al., 2019
High concentration NaOH solution immersing pretreatment	0.131/kg	Li et al., 2023
Alkaline peroxide pretreatment	0.212/kg	Wang et al., 2023
Autoclaving pretreatment	0.305/kg	Yeh et al., 2022
Combined pretreatment with ensiling and NaOH	0.103/kg	This study

### 3.4 Microbial community analysis

**Figure 5:**
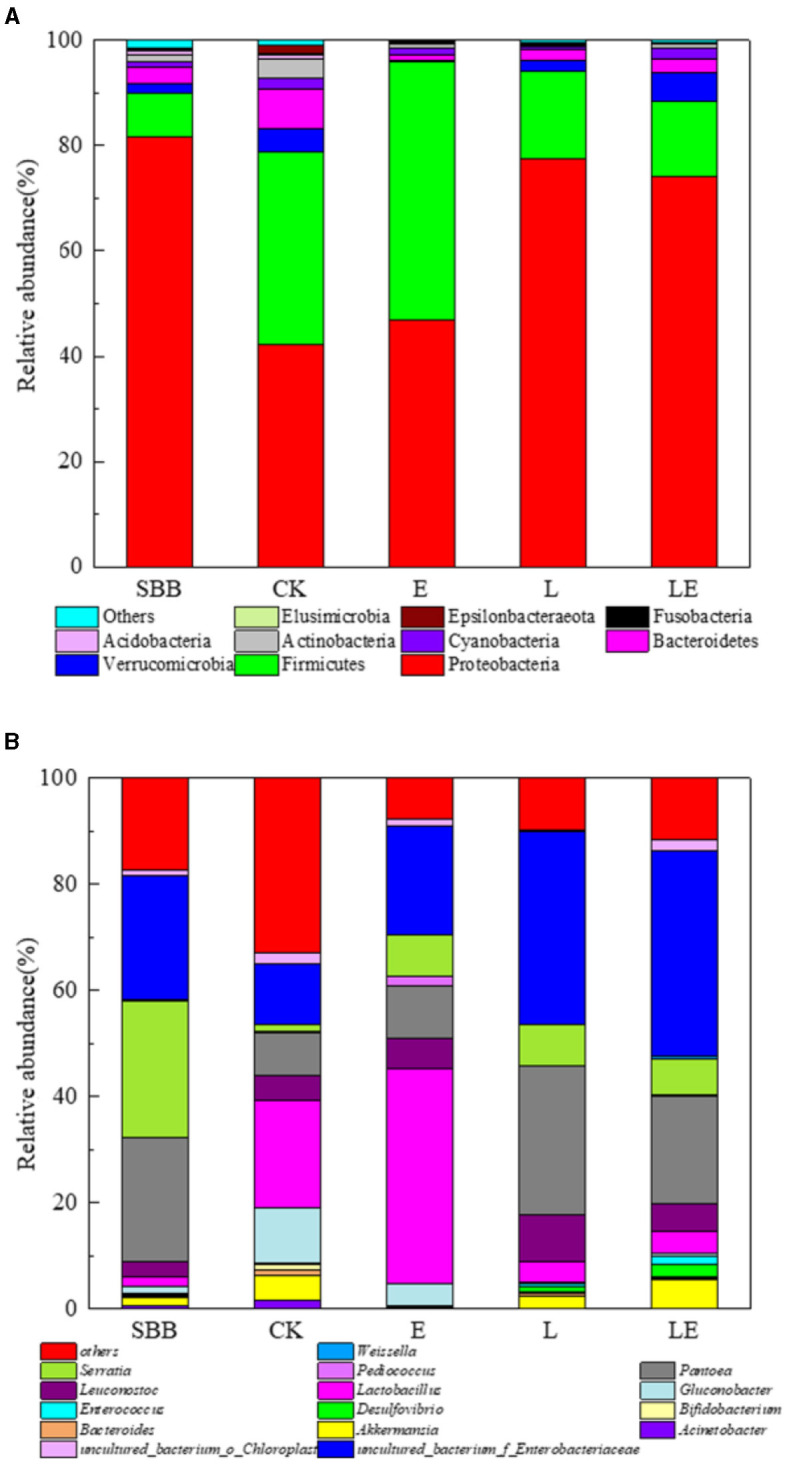
The relative abundance of the bacterial community at phylum level **(A)** and genera level **(B)** in sweet sorghum bagasse after combined treatment. SBB, sweet sorghum bagasse; CK, no additive control; E, ensiling enzyme; L, *Lactobacillus Plantarum*; LE, *L.plantarum*+ensiling enzyme.

The microbial community of SBB with different groups after combined treatment was analyzed. From the alpha diversity analysis (Table 2), the coverage rate of all samples is ~0.999, indicating that most of the bacteria have been identified. After treatment, the ACE and Chao index in the three additive groups decreased compared to raw SBB, while the CK group increased, indicating that the species richness of SSB decreased after combined pretreatment. The Shannon index and the PD of the whole tree index showed similar trends. The bacteria at the phylum level showed that the raw materials of SSB were mainly Proteobacteria (82.5%). After pretreatment, the dominant phyla in the four groups were Proteobacteria and Firmicutes, and the abundance of Proteobacteria decreased to varying degrees with the abundance of Firmicutes increased to varying degrees (Figure 5A). The abundance of Proteobacteria and Firmicutes in the LE group was 76.3 and 13.6%, respectively. At the genus level, the dominant bacteria in the raw materials of SSB were *Serratia, Pantoea*, and *uncultured_bacterium_f_Enterobacteriaceae* (Figure 5B). After combined treatment, all groups contained *Enterococcus, Lactobacillus, Leuconostoc*, and *Weisseria*. When compared with raw materials, the abundance of *Serratia* in the four groups decreased significantly after pretreatment with *uncultured_bacterium_f_Enterobacteriaceae* had a greater abundance. In addition, the total abundance of lactic acid bacteria in the four groups increased significantly after pretreatment compared with raw materials.

**Table 2:** Alpha-diversity indexes of different systems.

**Treatments**	**Reads**	**Feature**	**ACE**	**Chao1**	**Shannon**	**PD of whole tree**	**Coverage**
SBB	79,491a	422.00b	437.44b	445.42ab	3.92b	28.92b	0.9994b
CK	79,258c	432.20a	443.07a	448.05a	5.62a	29.49a	0.9996a
L	79,109d	353.20d	384.35d	395.06c	3.51c	25.39c	0.9993bc
E	79,254c	348.60e	377.56e	389.39d	3.33d	25.84c	0.9993bc
LE	79,369b	406.40c	426.75c	436.50b	3.91b	28.06b	0.9994b
Significance	<0.01	<0.01	<0.01	<0.01	<0.01	<0.01	<0.001

The different alphabetic letters means difference significance.

In this study, the microbial community of SBB after combined treatment was significantly varied. The alpha analyses such as ACE, Chao, and Shannon index results showed the microbial diversity of SBB decreased after combined treatment, which was consistent with the previous study (Wang et al., 2022). Similar to our study, Proteobacteria and Firmicutes were usually found to play main roles in anaerobic environments (Mu et al., 2020). Firmicutes is an important acid-hydrolyzing microbial group during anaerobic silage, which can produce a large number of extracellular enzymes, while Proteobacteria can digest organic matter (Ren et al., 2021). Similar results at the genus level were also observed in a previous study, where the dominant bacteria in the raw material of SSB reported were *Pantoea* (39%) and *Lactobacillus* (17.10%) (Dong et al., 2020). The reasons for slight difference may include factors such as climate, chemical composition of SSB, and region. The increased abundance of *Pantoea* might contribute to the decrease of ammonia-N given by the prior report (Ogunade et al., 2017). At the same time, after ensiling pretreatment, all groups contained *Enterococcus, Lactobacillus, Leuconostoc*, and *Weisseria*. These bacteria, as ideal lactic acid bacteria, randomly existed on the surface of silage, began lactic acid fermentation at the early stage of the silage process, and promoted pH reduction at the later stage (Luo et al., 2021).

### 3.5 Correlation analysis

**Figure 6:**
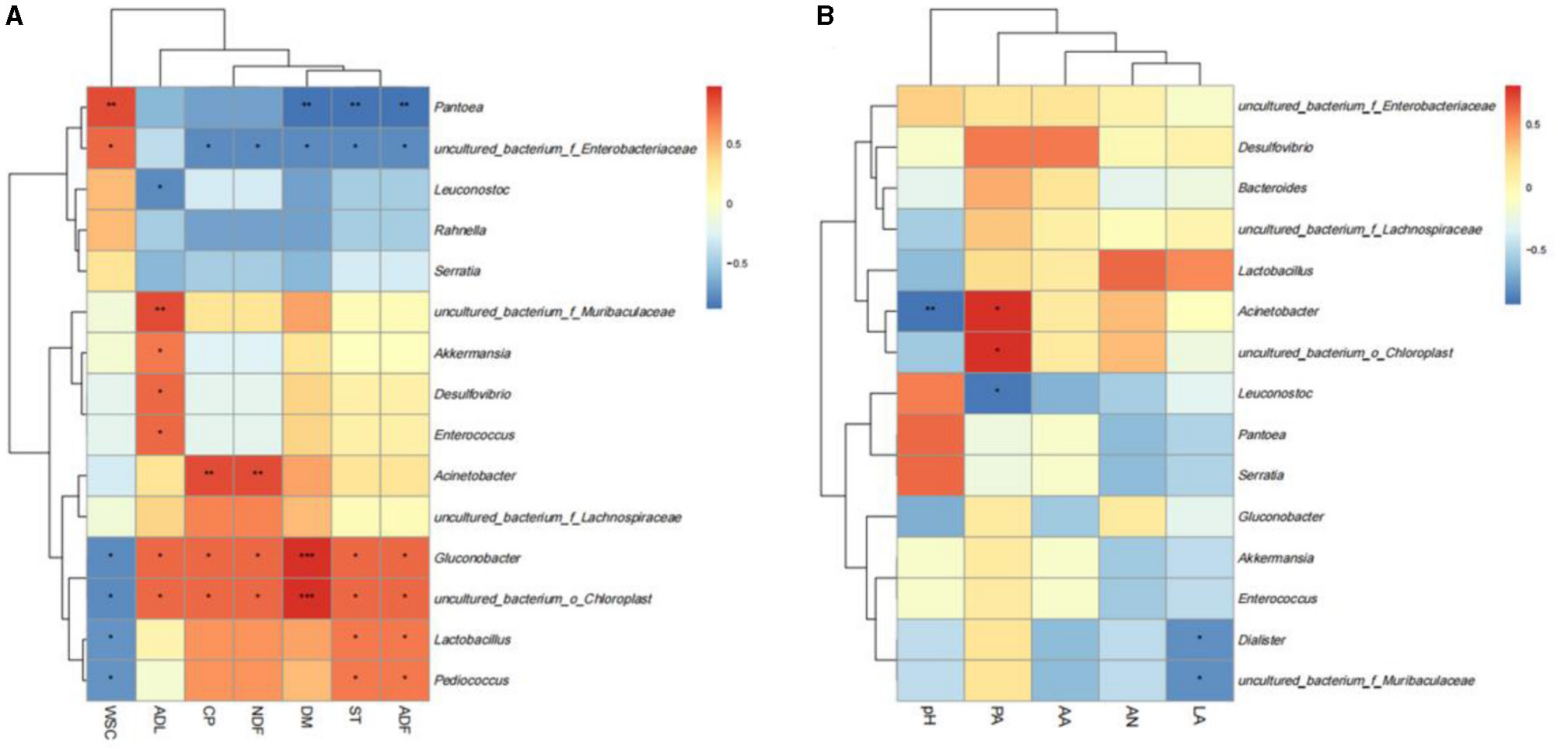
Correlation analysis between fermentation quality **(A)** and chemical components **(B)** with microorganisms. The red color means positively correction. ^*^p < 0.05; ^**^p < 0.01; ^***^p < 0.001.

To better reveal the relationship between chemical components and microbial kinetics during the silage of SSB, Spearman's correlation heat map at the genus level was used to analyze the correlation between bacteria and chemical components (Figure 6A). The correlation heatmap showed that CP was significantly positively correlated with *Acinetobacter, uncultured_bacterium_o_Chloroplast*, and *Gluconobacter*. The positive correlation between *uncultured_bacterium_o_Chloroplast* and PA was also consistent. After ensiling pretreatment, the content of WSC was negatively correlated with *Lactobacillus, Gluconobacter, uncultured_bacterium_o_Chloroplas*, and *Pediococcus*. In addition, ADL was positively associated with *Akkermansia, Desulfovibrio, Enterococcus, Gluconobacter*, and *uncultured_bacterium_o_Chloroplas*, but the relative abundance of these bacteria was low, suggesting that they may act synergistically to affect ADL content rather than individual bacteria genus function. *Pantoea* was negatively correlated with chemical components such as DM, CP, ST, and ADF and positively correlated with WSC. Additionally, a correlation analysis between fermentation characteristics and microbial flora was also conducted (Figure 6B). The results showed that LA was negatively correlated with *Dialister* and *uncultured_bacterium_o_Muribaculaceae*, PA was positively correlated with *Acinetobacter* and *uncultured_bacterium_o_Chloroplas*, and LA was negatively correlated with *Leuconostoc*.

It is well-known that the content of chemical components in raw materials is the main factor affecting silage (Dong et al., 2020). In this study, the correlation analysis was consistent with the previously reported effects of cellulase and *L. plantarum* on the silage quality of high-moisture amaranth and straw (Mu et al., 2020). There was showed the positively correlated with *Acinetobacter, uncultured_bacterium_o_Chloroplast*, and *Gluconobacter*, which were reported to be efficient acid-producing bacteria that can produce organic acids, thereby lowering pH and reducing protein loss (Ren et al., 2021). *Pantoea* was negatively correlated with chemical components such as DM, CP, ST, and ADF and positively correlated with WSC, which is consistent with previous findings (Ren et al., 2021). *Pantoea* is the most important epiphytic bacteria in the raw material of SSB. The epiphytic microbial flora in the raw material not only plays an important role in the natural silage fermentation but also affects the nutrient competition or interaction in the microbial ecosystem. The effectiveness of exogenous inoculants may also be the reason why the four groups contained a higher abundance of *Pantoea* after silage, and *Pantoea* was significantly associated with most chemical components. Moreover, microbial metabolism in the silage process can produce various organic acids, which can change the quality of silage by converting crude protein and other components into ammonia nitrogen. Lactic acid bacteria mainly inhibit the proliferation of undesirable microorganisms by producing lactic acid and reducing pH (Dong et al., 2020). Lactobacillus is an acid-tolerant bacterium, which may have needed some other methods like bacterial immobilization when combined using NaOH pretreated due to the high pH.

## 4 Conclusion

In this study, a combined pretreatment of silage and NaOH was applied to remove lignin from SSB and largely improve enzymatic hydrolysis. The results showed that the LE-2NaOH group had the highest efficiency of pretreatment. The combined pretreatment could increase ST and CP contents, decrease the pH, and increase the LA and AA contents, thereby improving silage quality and effective preservation of nutrient components, but also remove lignin and hemicellulose, thus altering the dense structure of SSB and improving the efficiency of enzymatic hydrolysis and saccharification. This study would provide a promising technology for enhancing biomass conservation and utilization.

## Data availability statement

The data presented in the study are deposited in the NCBI BioProject repository, accession number PRJNA1080473.

## Author contributions

SZ: Writing – original draft. HL: Writing – review & editing. TS: Project administration, Supervision, Writing – review & editing. AK: Methodology, Supervision, Writing – review & editing.
